# A Smart High Accuracy Silicon Piezoresistive Pressure Sensor Temperature Compensation System

**DOI:** 10.3390/s140712174

**Published:** 2014-07-08

**Authors:** Guanwu Zhou, Yulong Zhao, Fangfang Guo, Wenju Xu

**Affiliations:** State Key Laboratory for Manufacturing Systems Engineering, Xi'an Jiaotong University, No. 28, Xianning West Road, Xi'an 710049, China; E-Mails: alainzhou@stu.xjtu.edu.cn (G.Z.); erfang71@stu.xjtu.edu.cn (F.G.); xuwenju@stu.xjtu.edu.cn (W.X.)

**Keywords:** silicon piezoresistive pressure sensor, artificial neural networks, polynomial fitting, extreme learning machine, temperature compensation

## Abstract

Theoretical analysis in this paper indicates that the accuracy of a silicon piezoresistive pressure sensor is mainly affected by thermal drift, and varies nonlinearly with the temperature. Here, a smart temperature compensation system to reduce its effect on accuracy is proposed. Firstly, an effective conditioning circuit for signal processing and data acquisition is designed. The hardware to implement the system is fabricated. Then, a program is developed on LabVIEW which incorporates an extreme learning machine (ELM) as the calibration algorithm for the pressure drift. The implementation of the algorithm was ported to a micro-control unit (MCU) after calibration in the computer. Practical pressure measurement experiments are carried out to verify the system's performance. The temperature compensation is solved in the interval from −40 to 85 °C. The compensated sensor is aimed at providing pressure measurement in oil-gas pipelines. Compared with other algorithms, ELM acquires higher accuracy and is more suitable for batch compensation because of its higher generalization and faster learning speed. The accuracy, linearity, zero temperature coefficient and sensitivity temperature coefficient of the tested sensor are 2.57% *FS*, 2.49% *FS*, 8.1 × 10^−5^/°C and 29.5 × 10^−5^/°C before compensation, and are improved to 0.13%*FS*, 0.15%*FS*, 1.17 × 10^−5^/°C and 2.1 × 10^−5^/°C respectively, after compensation. The experimental results demonstrate that the proposed system is valid for the temperature compensation and high accuracy requirement of the sensor.

## 1. Introduction

Pressure is an important monitored parameter in industrial fields for process control and safety, so there is a massive demand for suitable pressure sensors. Because of their good accuracy, high sensitivity and excellent linearity, silicon piezoresistive MEMS pressure sensors are one of most reported and developed micromachined devices [1], and are widely used in various systems like automobiles, biomedical and process control systems for their low cost, small size and mature fabrication technology [2]. However, thermal drift caused by the inherent cross temperature sensitivity of silicon sensors [3] has a significant impact on the sensor accuracy, sensitivity and linearity. In order to reduce the impact of thermal drift, various temperature compensation techniques have been proposed, which mainly include three techniques: Hardware compensation, software compensation and hybrid approaches [4]. In addition, some fabrication processes and novel designs have also been used for temperature compensation.

Software methods have been widely applied as a digital approach for silicon sensor smart calibration systems because of their cost-effectiveness and high accuracy. Some calibration table methods based on look-up tables have been proposed as the simplest form of digital compensation [5]. However, the accuracy achieved by these methods is directly correlated to the used memory capacity. The IEEE1541.2 standard recommended a Taylor expansion as a general approach to describe sensor characteristics, but it requires a lot of calibration points for the temperature compensation. Šaponjić reported a microcontroller to ensure temperature compensation and linearization of the sensor using a second-order polynomial [6], which contributes to a better accuracy over a small measuring range. Zarnik *et al.* [7] used a rational polynomial approximation instead of Taylor expansion to represent the temperature variations of sensor characteristics by setting a seven-point calibration scenario. However, the accuracy the measured sensor achieved was about 0.4% *FS* in the compensation range 10 to75 °C.

Meanwhile, several software schemes based on artificial neural networks (ANNs) have been proposed to reduce the temperature effect on the accuracy of the applied pressure readout. Patra *et al.* [8,9] developed two approaches for capacitor pressure sensor modeling based on a functional link artificial neural network (FLANN) and back propagation neural network (BP), respectively, which achieve a low accuracy 3% *FS* over a wide temperature range of −50 ∼ 150 °C and a better accuracy of 1% *FS* in a temperature range of −20 ∼ 70 °C. Pramanik described an intelligent scheme using BP that obtained an error reduction of approximately 98% in the pressure range of 0 ∼ 1 bar and temperature range of 25 ∼ 80 °C [10]. Futane *et al.* [11] presented an CMOS analog ASIC design of a feed forward neural network for temperature drift compensation of a piezoresistive pressure sensor in which the error for compensated sensor was reduced to 0.1% in the temperature range of 0 ∼ 70 °C. However, ANN-based approaches were not clarified in the performances and configuration of neural networks.

Some hybrid approaches have also been reported, Chen presented a temperature compensation system which used a MAX1452 processor as the hardware compensation unit and a curve fitting algorithm based on a cubic B-spline [12], but the system is unsuitable for batch compensation. A MLX90257 absolute integrated pressure sensor adopts analog signal conversion to adjust the offset and span of a sensor by setting operational amplifiers using a 3-point temperature and 2-point pressure calibration [13]. Though it is a cost-effective temperature compensation method, the achieved accuracy (±1%) is not satisfactory. Overall, despite various approaches explored for the thermal drift of silicon sensors, many problems still exist, such as high cost, low accuracy and so on.

In this paper, a smart temperature compensation system is proposed to obtain high accuracy and facilitate batch fabrication, and a theoretical analysis of the relationship between the accuracy and thermal drift is conducted. An effective conditioning circuit is designed to process signals and acquire data. The hardware for implementing the system is fabricated, and a digital thermometer is integrated to sense ambient temperature. A program is developed on LabVIEW which incorporates an extreme learning machine (ELM) as the calibration algorithm for the pressure drift. Experiments, which take a microfabricated silicon pressure sensor as the compensated subject, are carried out to validate the effectiveness of designed system. Comparisons are done to evaluate the performances of the proposed ELM with other compensation algorithms. The compensated sensor aims at providing the pressure measurement of oil-gas pipelines to oil and gas fields within certain temperature range.

## 2. Temperature Effects and Temperature Compensation

### 2.1. Analysis of Temperature Effects on Accuracy

**Figure 1.**
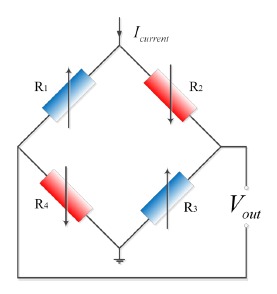
Schematic of the piezoresistive pressure sensor.

The piezoresistive pressure sensor design is based on piezoresistive effects which can convert physical signal into electrical signal. Generally, the piezoresistive pressure sensor consists of four piezoresistors (*R*_1_ = *R*_2_ = *R*_3_ = *R*_4_ = *R*) placed on the stress concentration regions in a Wheatstone bridge configuration. The equivalent circuit model of the pressure sensor is shown in Figure 1.

When the pressure sensor is driven by a constant current source *I_current_* and the four resistances satisfy the four conditions (see [14]), the output voltage of sensor can be expressed as:

(1) $$\begin{array}{ll} V_{\text{out}} & =\frac{\left(R_{2}+\Delta R\right)\times \left(R_{4}+\Delta R\right)-\left(R_{1}-\Delta R\right)\times \left(R_{3}-\Delta R\right)}{\left(R_{1}-\Delta R\right)+\left(R_{2}+\Delta R\right)+\left(R_{3}-\Delta R\right)+\left(R_{4}+\Delta R\right)}\times I_{\text{current}}\\ & =\Delta R\times I_{\text{current}}\\ & =I_{\text{current}}R\pi \sigma \end{array}$$

where, Δ*R* is resistance change of every of four piezoresistors, π is the piezoresistive coefficient of silicon, and σ is the stress applied on silicon.

It is found that there is a linear relationship between *V_out_* and σ from Equation (1) when both the piezoresistors *R* and piezoresistive coefficient π are constants. However, the change of ambient temperature has a great influence on the arm resistance of the Wheatstone bridge and the piezoresistive coefficient of silicon. The resistance and piezoresistive coefficient are defined as follows:

(2) $$\{\begin{array}{c} R=R_{0}(1+\alpha \Delta \text{T})\\ \pi =\pi_{0}(1+\beta \Delta \text{T}) \end{array}$$

where *R*_0_, π_0_ are the resistance of piezoresistors and piezoresistive coefficient of silicon at room temperature respectively, *α* and *β* are the temperature coefficient of piezoresistors and piezoresistive coefficient of silicon respectively, and Δ*T* is the variable temperature.

At the same time, a strain generates between the supporting beam and the substrate of pressure sensor due to the temperature variation. The variable quantity of residual stress can be represented as [15]:

(3) $$\Delta \sigma =\frac{\left(\alpha_{s}-\alpha_{g}\right)E_{0}\Delta T}{1+\mu }$$

where *α_s_*, *α_g_* are the thermal expansion coefficients of silicon and glass respectively, *E*_0_ is the temperature coefficient of silicon at Kelvin temperature. Substituting Equations (2) and (3) into Equation (1) yields:

(4) $$\begin{array}{ll} V_{\text{out}} & =I_{\text{current}}R\pi \sigma \\ & =I_{\text{current}}R_{0}(1+\alpha \Delta \text{T})\pi_{0}(1+\beta \Delta \text{T})\left(\sigma +\frac{\left(\alpha_{s}-\alpha_{g}\right)E_{0}\Delta \text{T}}{1+\mu }\right) \end{array}$$

In practical situation, Equation (4) needs to add the offset component *θ* caused by the error of fabrication process among the four resistors [16]. To express the output of sensor accurately, Equation (4) should be rewritten as follows:

(5) $$\begin{array}{ll} V_{\text{out}} & =I_{\text{current}}R_{0}(1+\alpha \Delta \text{T})\pi_{0}(1+\beta \Delta \text{T})\left(\sigma +\frac{\left(\alpha_{s}-\alpha_{g}\right)E_{0}\Delta \text{T}}{1+\mu }+\theta \right)\\ & =I_{\text{current}}R_{0}\pi_{0}\left[1+\left(\alpha +\beta +\alpha \beta \Delta \text{T}\right)\Delta \text{T}\right]\left(\sigma +\frac{\left(\alpha_{s}-\alpha_{g}\right)E_{0}\Delta \text{T}}{1+\mu }+\theta \right) \end{array}$$

From the above analysis, the sensor output *V_out_* is not proportional to the temperature variation Δ*T*. Moreover it can be seen in Equation (5) that α and β are correlated with *V_out_*. Both of them are the functions of doping concentration.

### 2.2. Temperature Compensation Algorithm

**Figure 2.**
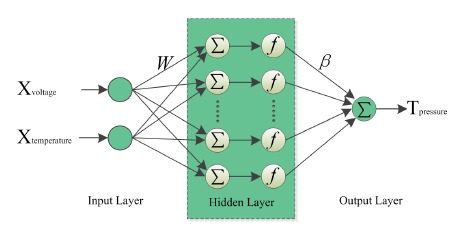
The structure of ELM.

Almost all pressure sensors based on silicon have linearity errors and temperature drift due to the physical properties of silicon. ANN not only can be used to reduce the influence of temperature, but also compensate the zero drift and linearity errors. The single hidden layer feed forward neural network (SLFN) which can approximate any continuous function in theory is currently one of the widely used network structures. A relatively novel learning algorithm called ELM [17] for SLFN (see Figure 2) is selected as the temperature compensation algorithm.

In this paper, the input variables are the digital signals of pressure and temperature corresponding to two neurons of input layer, and the output variable is pressure compensated by network. The relationship between the output *t_j_* and the input *x_j_* can be expressed using Equation (6):

(6) $$\begin{array}{cc} t_{j}=\overset{Ñ}{\underset{i=1}{\text{∑}}}\beta_{i}f\left(w_{i}x_{j}+b_{i}\right), & j=1\cdots ,N \end{array}$$

where *w_i_* and *β_i_* are the weight vectors connecting input-hidden layer and hidden-output layer, respectively; *b_i_* is bias of *i*th hidden neuron, *Ñ* is hidden node number, *N* is the number of sample, and *f* is a nonlinear activation function.

**Figure 3.**
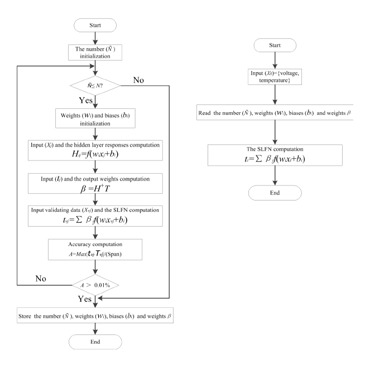
The temperature compensation flowchart of ELM. (**a**) Temperature calibration flowchart using SLFN trained by ELM; (**b**) Temperature compensation flowchart using the trained SLFN.

In ELM, *w_i_* and *b_i_* are randomly generated in the range of 0 to 1, and *β_i_* is calculated with matrix operations using Equation (7):

(7) $$\beta =H^{+}T$$

where *H* = {*h_i,j_*,*j* = 1, …,*N*;*i* = 1, …Ñ} is the hidden-layer output matrix,*T* = [*t*_1_, …, *t_N_*]*^T^*, *h_i,j_* = *f*(*w_i_x_j_*+*b_i_*) denotes the output of the *i*th hidden neuron with respect to *x_i_*, *H*^+^ is the Moore Penrose generalized inverse of the matrix *H* and *H*^+^ = (*H^T^H*)^−1^ *H^T^*. To improve the generalization of ELM, the flowchart of ELM for temperature compensation is designed as shown in Figure 3.

In the compensation process, the required number of hidden nodes can be determined by the flowchart of Figure 3a according to Theorem 2.2 in [17]. ELM learns faster with a higher generalization and operability than the traditional learning algorithms for SLFN such as gradient-based learning methods. In order to evaluate the effectiveness of ELM, other methods such as BP, radius basis function (RBF) neural network, support vector machine (SVM) [18], variable coefficient regression (VCR) [19] and the rational polynomial approximation algorithm (RPA) are selected to compare with ELM.

## 3. Design of the Smart Temperature Compensation System

According to the analysis discussed in Section 2, it is found that the relationship of *V_out_* and Δ*T* is nonlinear. Considering the features mentioned above, the relationship between the accuracy and temperature also is nonlinear. Therefore, a smart temperature compensation system is proposed here. In the system, a membrane type piezoresistive pressure sensor is adopted; the relevant designs and experiments are carried out for high accuracy. This proposed approach would be useful for other types of sensors such as resistance strain sensor, capacitive sensor, piezoelectric sensors, *etc.*

### 3.1. Hardware Design of the System

**Figure 4.**
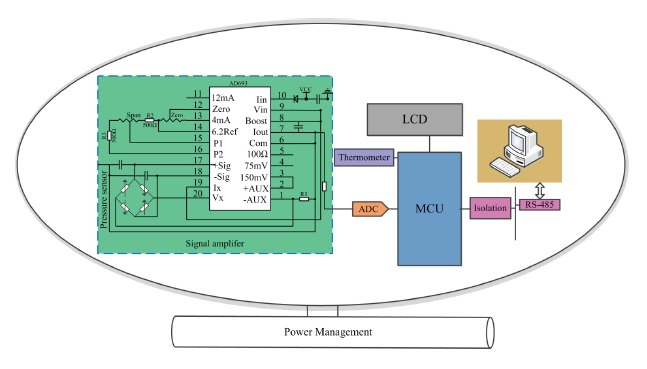
Schematic of system hardware.

The hardware comprises following modules: pressure and temperature sensors, signal conditioning module, MCU, liquid crystal display (LCD), communication module, power supply module, as shown in Figure 4.

**Figure 5.**
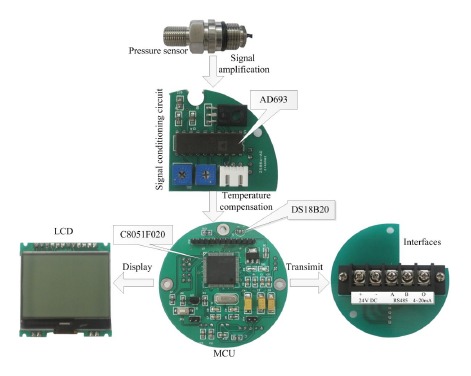
Hardware components of system.

In the circuit, the AD693 is selected as the front-end amplifier to replace the traditional discrete designs, which provide excitation current and output current signals of 4 ∼ 20 mA for pressure sensor. Compared with the traditional amplification circuit design, the AD693 can make the design of signal conditioning easier. Its applied circuit diagram with 0 ∼ 30 mV unipolar input and 4 ∼ 20 mA output is shown in Figure 4. DS18B20 is selected as the digital thermometer. A C8051F020 chip developed by Silicon Lab (Austin, Texas, US) is selected as controller which is generally more than 10 times as fast as the 80C51 series in instruction execution speed, and integrates an analog-to-digital-converter (ADC) and digital-to-analog-converter (DAC). It has a peak throughput of 25 MIPS and 12-bit ADC with the power consumption of about 4 mW. These advantages meet the requirement of high accuracy and simplify the circuit at the same time. The RS485 interface circuit can output digital signal and communicate with a personal computer (PC). Figure 5 shows the system hardware.

### 3.2. Software Design of the System

**Figure 6.**
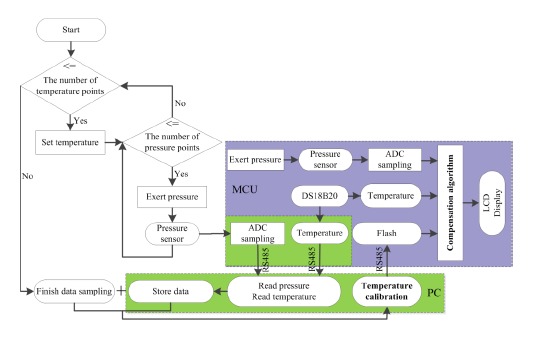
The flowchart of the temperature compensation system.

LabVIEW 9.0 is used to design graphical user interface (GUI) of temperature compensation system and write digital data generated by calibration into the microcontroller via a USB-RS485 communication adapter. Figure 6 shows the flowchart of the pressure measurement system. The PC is used for temperature calibration, that is, determination of the number of hidden nodes and the output weights of SLFN trained by ELM. Then the trained SLFN is ported to the MCU. The MCU acquires the object pressure and ambient temperature, and calculates the compensated pressure using the ported SLFN. Finally, all the measured information is displayed on the LCD.

## 4. Experimental Setup and Results

### 4.1. Calibration Setup

The specific steps of temperature compensation using ELM are as follows:

Step 1 Normalize the sample data into the range [−1, 1], which is measured within a pressure range from 0 to 20 MPa within a temperature range from −40 to +85 °C;
Step 2 Randomly divide the normalized sample data (voltage temperature, applied pressure) into training data and testing data with a ratio of 2 to 1;
Step 3 Orderly select the number of hidden nodes from 1 to the number of training samples;
Step 4 Initialize the input weights and and hidden layer biases randomly, input training data and compute the output weights of SLFN;
Step 5 Based on weights and biases obtained by Step 4, compute the outputs of the testing data;
Step 6 Repeat Steps 2–4, until a satisfactory compensation accuracy is obtained;
Step 7 Write the weights and biases of SLFN into microprocessor, verify the algorithm within a pressure range from 0 to 20 MPa within −40 ∼ 85 °C;
Step 8 Calculate the real accuracy of calibrated sensor.

The weights and biases of trained neural network for temperature compensation are programmed into the microcontroller. The number of calibration points and temperature stabilization time represent the limiting factor to calibration capacity. In order to test the calibration capacity sufficiently and select the suitable number of calibration points for high accuracy using ELM, three configurations of calibration points are designed as shown in Table 1.

**Figure 7.**
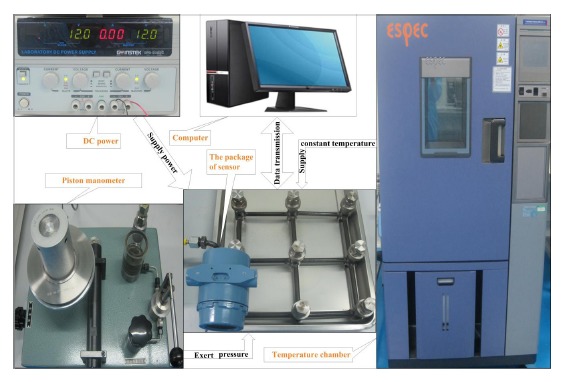
The experiment setting of the system.

The experiment process of temperature compensation is shown in Figure 7, which comprises 24 V DC power, temperature test chamber and piston manometer. First, the system hardware is put into a temperature test chamber. The temperature test chamber is used to apply the specified temperature to the pressure sensor and keep the temperature stabilized for one hour. Then the piston manometer exerts different pressures on the pressure sensor according to the experiments. The computer is operated manually to read the pressure and temperature data from the MCU in a wired communication way when each pressure point exerted is stable. At last, the trained SLFN for temperature compensation is ported to the MCU after temperature calibration in the PC.

### 4.2. Calibration Results and Discussion

**Figure 8.**
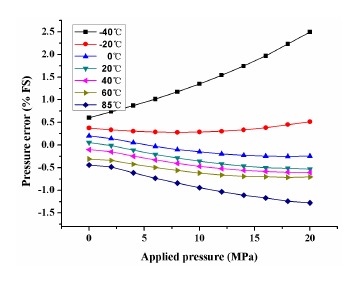
Pressure error in percentage between linear fit and actual characteristics.

In order to improve the sensor's accuracy, the static pressure was measured within a pressure range of 0 ∼ 20 MPa with a 2 MPa step within a temperature range of −40 ∼ 85 °C with a 20 °C step. The experimental data is fitted using the least square method. The pressure error in percentage between linear fit and actual data is shown in Figure 8, which shows a poor linearity of 2.5% *FS* which is mainly caused by the thermal drift.

**Figure 9.**
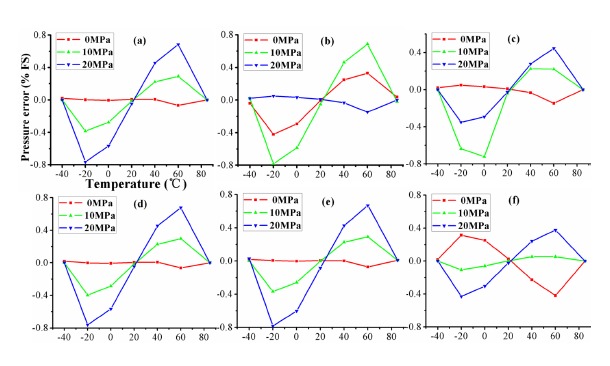
The compensated errors of algorithms at three pressure points on temperature using 3 × 3 calibration: (**a**) VCR; (**b**) RPA; (**c**) BP; (**d**) SVM; (**e**) RBF and (**f**) ELM.

The compensated pressure error was determined for all points at which pressure and temperature were measured. In this 3 × 3 configuration experiment, nine samples are used as the experimental data. Figure 9 shows the compensated error of algorithms on temperature at three pressure points using the 3 × 3 calibration. It is seen from Figure 9 that the compensated errors of all algorithms for three pressure of 0, 10 and 20 MPa are basically similar, in which the maximum error is about 0.8% *FS* due to the small samples. The experimental comparisons for VCR, RPA, BP, SVM, RBF and ELM are given in Table 2. From Table 2, the performances of sensor calibrated by algorithms are the same when considering the accuracy, linearity, zero temperature coefficient, and sensitivity temperature coefficient. The mean squared error (MSE) of ELM is 1.6 × 10^−3^ between the compensated pressure and actual pressure. These configured parameters of algorithms in 3 × 3 configuration are shown in Table 3.

**Figure 10.**
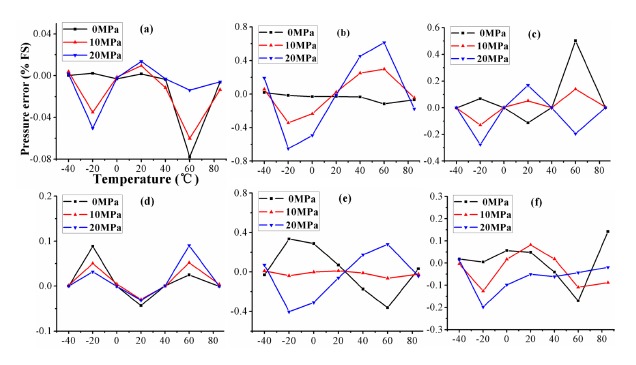
The compensated errors of algorithms at three pressure points on temperature using 4 × 6 calibration: (**a**) VCR; (**b**) RPA; (**c**) BP; (**d**) SVM; (**e**) RBF and (**f**) ELM.

In this 4 × 6 configuration experiment, 24 samples are used as the experimental data. Figure 10 shows the compensated error at the three input pressure as a function of temperature within the full measurement range for compensated sensors. It can be found from Figure 10 that the compensated errors of VCR, RPA, BP, SVM, RBF and ELM are reduced remarkably compared with errors in Figure 9. VCR obtains the minimum compensated error 0.08% *FS* at three pressure inputs. However, the experimental comparisons for VCR, RPA, BP, SVM, RBF and ELM in Table 4 show the sensor compensated by ELM has better accuracy and nonlinearity. The accuracy and nonlinearity are improved to 0.23% *FS* and 0.3% *FS* in Table 4 using 4 × 6 calibration from 0.8% *FS* and 0.6% *FS* in Table 2 using 3 × 3 calibration. These configured parameters of algorithms in 4 × 6 configuration are shown in Table 5. VCR acquires the minimum MSE 1.5 × 10^−4^, which shows the fluctuation of errors is much less than in the other compensation results. These configured parameters of algorithms in 4 × 6 configuration are shown in Table 5.

**Figure 11.**
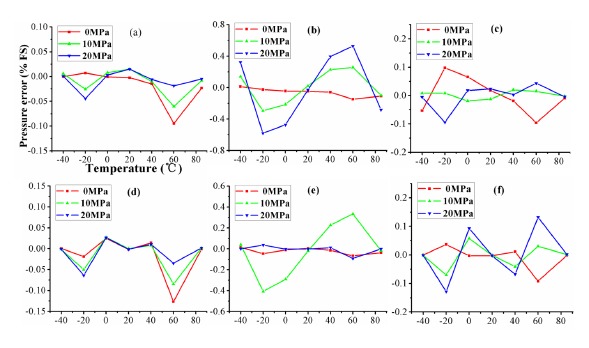
The compensated errors of algorithms at three pressure points on temperature using 5 × 11 calibration: (**a**) VCR; (**b**) RPA; (**c**) BP; (**d**) SVM; (**e**) RBF and (**f**) ELM.

In this 5 × 11 configuration experiment, 55 samples are used as the experimental data. It can be found from Figure 11 that the compensated errors of ELM at three pressure points are reduced to 0.13% *FS*. Although VCR has the minimum error 0.1% *FS* at three pressure points, the experimental comparisons of algorithm in Table 6 show the sensor compensated by ELM obtains the best accuracy, nonlinearity and MSE. The accuracy, nonlinearity and MSE are 0.13% *FS*, 0.15% *FS* and 0.85 × 10^−4^, respectively. These configured parameters of algorithms in 5 × 11 configuration are shown in Table 7.

**Figure 12.**
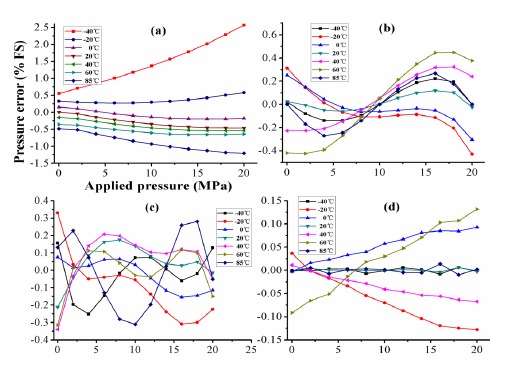
Compensated pressure errors of ELM: (**a**) before and (**b**) after compensation using 3 × 3 calibration; (**c**) after compensation using 4 × 6 calibration; (**d**) after compensation using 5 × 11 calibration.

Figure 12 compares the compensated error of ELM at full measurement range using different calibration configurations. It is obvious that the 5 × 11 calibration configuration has better performances as shown in Figure 12d. The compensated errors has smaller fluctuation on temperature in Figure 12d than others in Figure 12b,c. At the same time, the compensated error is about 2.5% *FS* before compensation, and is reduced to about 0.1% *FS* after ELM compensation.

According to Equation (5) in Section 2.1, the relationship between voltage and temperature is a third-order polynomial under ideal conditions, so all compensation algorithms used in the paper can approximate the polynomial in theory. However, it is difficult to get the ideal expression because of the fabrication limitations. From Tables 2 and 3, it can be observed that VCR and RPA with their operability and fast computation capability are suitable for temperature compensation when a few samples are available, but they can't provide as good accuracy as other algorithms. With the increase in sample numbers, the accuracy of sensor compensated by algorithms is improved which can be seen from Tables 2 and 6. It is deduced that sample number has a great influence on the algorithm performance. It takes a lot of time to set the reasonable parameters for BP, RBF and SVM for compensation due to the lack of theoretical guidance, which makes it difficult to achieve high accuracy. Thus, BP, RBF and SVM are suboptimal solutions for compensation of silicon sensors. Compared with them, ELM can provide higher accuracy, and is more suitable for compensation due to its low computational complexity and single-parameter setting with theoretical guidance.

## 5. Conclusions

A smart temperature compensation system is presented in this study. The implemented circuitry is structurally simple and suitable for batch fabrication. ELM with its single-parameter setting and fast learning speed is selected as the temperature compensation algorithm for piezoresistive silicon sensors. In order to test the effectiveness of the system, experiments are performed within 0 ∼ 20 MPa and −40 ∼ 85 °C. Three calibration point configurations are designed for high accuracy. To compare the calibration performance of ELM, VCR, RPA, BP, SVM and RBF are chosen. The experimental results indicate that ELM is a better compensation algorithm in terms of higher accuracy when using a 5-point temperature and 11-point pressure calibration. ELM can find the suitable SLFN structure for temperature compensation without intervention during calibration. The accuracy, linearity, zero temperature coefficient and sensitivity temperature coefficient of sensor calibrated by ELM are improved from 2.57% *FS*, 2.49% *FS*, 8.1 × 10^−5^/°C and 29.5 × 10^−5^/°C to 0.13% *FS*, 0.15% *FS*, 1.17 × 10^−5^/°C and 2.1 × 10^−5^/°C, respectively. The compensated pressure sensors can be applied for the pressure measurement of pipelines in the oil and chemical industry. In future work, we will focus on the overall compensations for hysteresis and accuracy with the appropriate software algorithms.

## Figures and Tables

**Table 1.** The configurations of calibration points.

**Configuration**	**Calibration Points**
3 × 3	(−40, 22.5, 85) °C
	(0, 10, 20) MPa

4 × 6	(−40, 0, 40, 85) °C
	(0, 4, 8, 12, 16, 20) MPa

5 × 11	(−40, −10, 20, 50, 85) °C
	(0, 2, 4, 6, 8, 10, 12, 14, 16, 18, 20) MPa

**Table 2.** Performances of sensor calibrated by algorithms using 3 × 3 calibration.

**Algorithms**	**Accuracy *A* (%*FS*)**	**Linearity δ*_l_* (%*FS*)**	**MSE**	**Temperature Coefficient of Zero *a*_0_ (/°C)**	**Temperature Coefficient of Sensitivity *a*_s_ (/°C)**
VCR	0.76	0.73	3.2 × 10^−3^	0.82 × 10^−5^	11.49 × 10^−5^
RPA	0.78	0.74	3.5 × 10^−3^	1.03 × 10^−5^	11.67 × 10^−5^
BP	0.78	0.67	4.8 × 10^−3^	1.65 × 10^−5^	6.40 × 10^−5^
SVM	0.85	0.88	4.3 × 10^−3^	1.76 × 10^−5^	22.91 × 10^−5^
RBF	0.76	0.86	3.7 × 10^−3^	0.76 × 10^−5^	11.42 × 10^−5^
ELM	0.76	0.55	1.6 × 10^−3^	5.84 × 10^−5^	6.43 × 10^−5^

**Table 3.** Configured parameters of algorithms using 3 × 3 calibration.

**Algorithms**	**Configured Parameters**
VCR	two second-order polynomials
RPA	two-dimensional rational
BP	structure: 2 × 4 × 1
SVM	‘−c 1.3 −g 0.2 −s 4’
RBF	structure: 2 × 9 × 1; Spread: 7.5
ELM	structure: 2 × 9 × 1

**Table 4.** Performances of sensor calibrated by algorithms using 4 × 6 calibration.

**Algorithms**	**Accuracy *A* (%*FS*)**	**Linearity δ*_l_* (%*FS*)**	**MSE**	**Temperature Coefficient of Zero *a*_0_ (/°C)**	**Temperature Coefficient of Sensitivity *a*_s_ (/°C)**
VCR	0.32	0.29	1.5 × 10^−4^	0.83 × 10^−5^	2.91 × 10^−5^
RPA	0.65	0.66	27 × 10^−4^	1.17 × 10^−5^	10.05 × 10^−5^
BP	0.50	0.67	5.7 × 10−4	5.0 × 10−5	3.56 × 10−5
SVM	0.40	0.39	9.0 × 10^−4^	5.57 × 10^−5^	5.47 × 10^−5^
RBF	0.38	0.86	2.7 × 10^−4^	1.15 × 10^−5^	3.27 × 10^−5^
ELM	0.23	0.23	3.4 × 10^−4^	2.49 × 10^−5^	3.34 × 10^−5^

**Table 5.** Configured parameters of algorithms using 4 × 6 calibration.

**Algorithms**	**Configured Parameters**
VCR	two third-order polynomials
RPA	two-dimensional rational
BP	structure: 2 × 5 × 1
SVM	‘−c 1.7 −g 1.2 −s 4’
RBF	structure: 2 × 24 × 1; Spread: 6.5
ELM	structure: 2 × 11 × 1

**Table 6.** Performances of sensor calibrated by algorithms using 5 × 11 calibration.

**Algorithms**	**Accuracy *A* (%*FS*)**	**Linearity δ*_l_* (%*FS*)**	**MSE**	**Temperature Coefficient of Zero *a*_0_ (/°C)**	**Temperature Coefficient of Sensitivity *a*_s_ (/°C)**
VCR	0.32	0.29	1.5 × 10^−4^	0.98 × 10^−5^	2.89 × 10^−5^
RPA	0.60	0.62	24 × 10^−4^	1.36 × 10^−5^	8.98 × 10^−5^
BP	0.28	0.25	1.7 × 10^−4^	1.55 × 10^−5^	2.49 × 10^−5^
SVM	0.41	0.41	9.0 × 10^−4^	5.62 × 10^−5^	5.91 × 10^−5^
RBF	0.22	0.21	0.88 × 10^−4^	1.21 × 10^−5^	2.23 × 10^−5^
ELM	0.13	0.15	0.85 × 10^−4^	1.17 × 10^−5^	2.08 × 10^−5^

**Table 7.** Configured parameters of algorithms using 5 × 11 calibration.

**Algorithms**	**Configured Parameters**
VCR	two third-order polynomials
RPA	two-dimensional rational
BP	structure: 2 × 5 × 1
SVM	‘−c 1.2 −g 0.6 −s 4’
RBF	structure: 2 × 37 × 1; Spread: 3.5
ELM	structure: 2 × 30 × 1
